# Thymoquinone Effect on Monocyte-Derived Macrophages, Cell-Surface Molecule Expression, and Phagocytosis

**DOI:** 10.3390/nu14245240

**Published:** 2022-12-08

**Authors:** Nuha A. Alkhattabi, Sowsan A. Hussein, Nesrin I. Tarbiah, Reem Y. Alzahri, Reham Khalifa

**Affiliations:** 1 Biochemistry Department, Faculty of Science, King Abdulaziz University, Jeddah 21589, Saudi Arabia; 2 Department of Biology, College of Science, University of Jeddah, Jeddah 21493, Saudi Arabia; 3 Medical Microbiology and Immunology, Faculty of Medicine, Ain Shams University, Cairo 11566, Egypt

**Keywords:** *Nigella sativa*, thymoquinone, macrophages, surface molecules, phagocytosis, cytokines

## Abstract

Macrophages are one of the most important cells in the immune system. They act as links between innate and adaptive immunities. In this study, the aim was to examine thymoquinone effects on the immunological properties of different macrophages. Peripheral blood mononuclear cells were isolated from blood from healthy volunteers by negative selection of monocytes that had been cultured for seven days to differentiate into macrophages. Cells were cultured with or without the presence of thymoquinone (TQ), which was used in two different concentrations (50 μg/mL and 100 μg/mL. Cluster of differentiation 80 (CD80), cluster of differentiation 86 (CD86), and human leukocyte antigen DR isotype (HLA-DR) were measured by flow cytometry, and the secretion of interferon gamma (IFN-γ) and tumour necrosis factor alpha (TNF-α) was measured. Cells were also tested for their *E. coli* phagocytosis abilities. The data showed that the expression of HLA-DR was significantly higher in cells treated with 100 μL/mL TQ. In addition, IFN-γ concentration increased in the 100 μg/mL TQ-treated cells. The macrophage phagocytosis results showed a significant difference in 50 μg/mL TQ-treated cells compared to the controls. TQ may enhance the immunological properties of macrophages during the early stages of innate immunity by activating phagocytosis ability and by increasing the expression of HLA-DR and the secretion of IFN-γ, which may enhance the antigen-presentation capabilities of macrophages.

## 1. Introduction

Macrophages are a type of immune cell that play a defensive role against foreign pathogens. They play an essential role in the body’s homeostatic maintenance by removing interior waste materials and repairing tissues through phagocytosis, which plays a primary role in macrophage functionality within the immune system. Macrophages are found in all vertebrate tissues, and various stimuli may have different effects on macrophage phenotypes [1]. Two main macrophage lineages are known: first, the macrophages that differentiate from blood-circulating monocytes that arise from bone marrow myeloid progenitor cells and migrate to areas of host injury; and second, those that come from the yolk sac and are resident in tissues [2,3]. Macrophages, dendritic cells and B cells act as professional antigen-presenting cells (APCs) that present antigens and activate T cells via their cluster of differentiation—CD80, CD86, and major histocompatibility complex class II (MHC II)—leading to adaptive immune system activation [4,5,6,7]. Moreover, in addition to their role in phagocytosis, macrophages secrete a number of cytokines that induce immune-cell activity and activate a cascade of events during immune response. Two of the most important cytokines that are involved in the development of the pathogenesis of various diseases are interferon gamma (IFN-γ) and tumor necrosis factor alpha (TNF-α) [8,9]. During innate immunity, IFN-γ secreted by macrophages leads to the recruitment of neutrophils and T cells to the site of infection and promotes the killing of target cells through the activation of a Th1 response [10]. In addition, IFN-γ secreted by other cells, such as NK and T cells, activates macrophages and increases their production of various inflammatory molecules, including TNF-α. Thus, TNF-α is a potent proinflammatory cytokine that plays an important role in orchestrating inflammatory responses affecting many calls and organs and controls the release of other cytokines and chemokines [11].

Medicinal plant use has significantly increased in recent years, compared to the use of chemical drugs, for many reasons: plants are low in cost, easy to obtain without a prescription, and require no consultation with a healthcare professional; furthermore, people believe that treatments with natural products have fewer side effects than chemical treatments [12,13].

A herbal plant that has proved to enhance the activity of macrophages is *Nigella sativa* (*N. sativa*), also called black seed [14]. *N. sativa* seeds and their oil are commonly used to treat different diseases throughout the world [15]. The main abundant component of *N. sativa* seeds is thymoquinone (TQ), and most properties of *N. sativa* fundamentally result from TQ [16]. Several medicinal actions of TQ, involving anti-inflammatory, antioxidant, immunomodulatory, and antimicrobial effects, have been examined [17]. It is suggested that TQ controls proinflammatory cytokines, nitric oxide (NO) and myeloperoxidase [18]. In addition, the role of TQ in affecting both innate and adaptive immunity is explained by its action on the nuclear factor kappa beta (NF-kβ) signaling pathway that controls the expression of different receptors and the secretion of various immune modulators as proinflammatory cytokines and chemokines, such as TNF-α, interleukin-1 (IL-1), interleukin-6 (IL-6), interleukin-8 (IL-8), interleukin-18 (IL-18), macrophage inflammatory protein (MIP), monocyte chemoattractant protein (MCP), and other proteins involved in antigen presentation [19]. TQ was also found to have a role in controlling the Janus kinase/signal transducer and activator of transcription (JAK/STAT) signaling pathway, and thus affects inflammatory responses through different cytokines and growth factors. JAK/STAT is an intracellular pathway that controls different cell aspects such as differentiation, proliferation, communication migration and apoptosis [20]. This makes TQ an interesting therapeutic target in the treatment of different inflammatory and autoimmune diseases.

Studies on *N. sativa* effects on macrophages are limited. For that reason, we aimed to examine the effect of TQ, as the important component of *N. sativa*, on the immunological properties of macrophage cells by examining the effect of TQ on the expression of important macrophage surface molecules, on cytokine production, and on phagocytic ability.

## 2. Materials and Methods

### 2.1. PBMC Isolation and Separation of CD14^+^ Monocytes

Peripheral blood mononuclear cells (PBMCs) were isolated from 50 mL blood samples, in ethylenediamine tetraacetic acid (EDTA)-coated tubes, using Histopaque 1077 solution (Sigma Aldrich, Saint Louis, MO, USA) and density gradient centrifugation. Briefly, the 50 mL sample was diluted with 50 mL of phosphate-buffered saline (PBS) (20012043, Thermo Fisher, Waltham, MA, USA), and then 30 mL of the mixture was added to the already-aliquoted 10 mL Histopaque. After that, samples were centrifuged at 700× *g* for 30 min. The layer of PBMCs was extracted carefully, washed using PBS, and centrifuged at 350× *g* for 10 min. This washing step was repeated twice.

A monocyte isolation kit (Pan Monocyte Isolation Kit, human isolate classical (CD14^++^ CD16^−^), nonclassical (CD14^+^ CD16^++^), and intermediate (CD14^++^ CD16^+^) monocytes—Miltenyi Biotec, Bergisch Gladbach, Germany, catalog no. 130-096-537) was used, following the manufacturer’s instructions, to separate CD14^+^ monocytes, and the purity of the isolated cells was checked via flow cytometry.

### 2.2. Thymoquinone Preparation

Thymoquinone (274666-1G, Sigma-Aldrich, Saint Louis, MO, USA) was dissolved in RPMI 1640 medium (31870074, ThermoFisher, Waltham, MA, USA) containing 1% dimethyl sulfoxide anhydrous, ≥99.9% (276855, Sigma-Aldrich, Saint Louis, MO, USA). Two dilutions of the stock, 50 and 100 μg/mL, were prepared using RPMI 1640 medium.

### 2.3. Culturing Isolated Monocytes

CD14^+^-isolated monocytes were cultured in RPMI 1640 supplemented with 20% FBS (16140071, ThermoFisher, Waltham, MA, USA) and 10% penicillin/streptomycin (10378016, ThermoFisher, Waltham, MA, USA) in a 12-well plate as 5 × 10^5^ cells/mL/well in the presence of granulocyte-macrophage colony-stimulating factor granulocyte-macrophage colony stimulating factor (GM-CSF) (PHC2015, ThermoFisher, Waltham, MA, USA) at a final concentration of 10 ng/mL. Cells were incubated for 7 days at 37 °C and the media were changed on the third day. On day 7, differentiated macrophages were prepared for TQ treatment. For that, cells were prepared as controls, or treated with 50 μg/mL TQ or 100 μg/mL TQ, and incubated for 24 h. Cells were collected to analyze the expression of cell-surface molecules, CD80-PE (IM 1976, Beckman Coulter, Brea, CA, USA), CD86 (B7-2), –PE (IM2729U, Beckman Coulter, Brea, CA, USA), HLA-DR-APC (IM3653, Beckman Coulter, Brea, CA, USA), and CD14-FITC (B36297, Beckman Coulter, Brea, CA, USA) via flow cytometry using FACS Aria 3 from the BD company (Franklin Lakes, NJ, USA) followed by data analysis using FACSDiva version 9 software (BD Life Sciences, San Jose, CA, USA).

### 2.4. Phagocytosis Analysis

Control cells and cells treated with TQ 50 μg/mL or 100 μg/mL for 24 h were checked for their phagocytic functionality using the Green *E. coli* BioParticles™ Phagocytosis Kit (P35381, Thermo Fisher, Waltham, MA, USA). Following the kit instructions, the percentages of macrophage phagocytosis were measured by flow cytometry.

### 2.5. Cytokine Analysis by Enzyme-Linked Immunosorbent Assay (ELISA)

A human interferon-gamma enzyme-linked immunosorbent assay (ELISA) Kit (E0105Hu, Elabscience, Houston, TX, USA) and a human tumor necrosis factor-alpha ELISA Kit (E-EL-H0109, Elabscience, Houston, TX, USA) were used and the manufacturer’s instructions were followed.

### 2.6. Statistical Analysis

Data were analyzed using Statistical Package for the Social Sciences software version 25 (SPSS, IBM Corp., Armonk, NY, USA) and expressed as (mean ± standard deviation (SD)). An analysis of variance test was used to examine the difference between the three groups, while an unpaired *t*-test was used for the comparison of two groups as needed; *p* ≤ 0.05 was considered significant.

## 3. Results

### 3.1. Gating of CD14^+^ Monocytes and Purity Test

**Figure 1:**
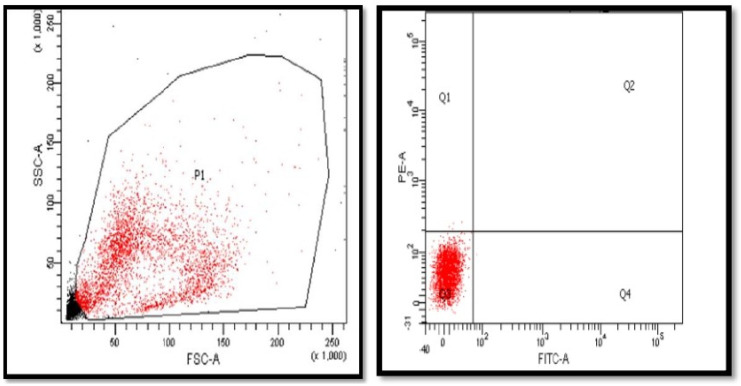
Gating of magnetic isolated monocytes from whole PBMCs in P1 gate. Following the isolation of PBMCs, monocytes were separated with negative selection and the population of separated cells identified using forward- and side-scattered laser beam. The monocytes that are presented in gate P1 were applied to the rest of the results. *n* = 8. PBMCs, peripheral blood mononuclear cells; SSC-A, side scatter paremeter; FSC-A, forward scatter parameter; PE-A, phycoerythrin; FITC-A, fluorescein isothiocyanate; Q1, quadrant 1; Q2, quadrant 2; Q3, quadrant 3; Q4, quadrant 4.

**Figure 2:**
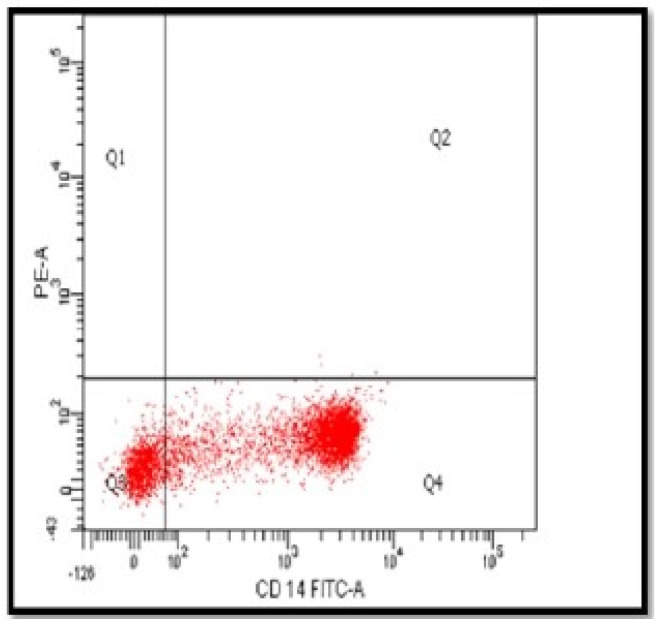
The purity of CD14^+^-separated monocytes. After negative selection of monocytes, isolated cells were stained using extracellular staining CD14-FITC. The purity of separated monocytes ranged from 83 to 89%. CD14-FITC-A, fluorescein isothiocyanate.

Using a negative magnetic monocyte isolation kit, the population of the isolated cells was identified with the forward and side scatter of the flow cytometer. The monocyte cell population has been gated in P1 (Figure 1). The average number of monocytes was (0.8 × 10^7^)/50 mL blood. The purity of monocytes CD14^+^ was checked using extracellular staining of the cells with CD14-FITC with purity arranged from 83% to 89% (Figure 2).

### 3.2. Differentiation of Monocytes to Macrophages

**Figure 3:**
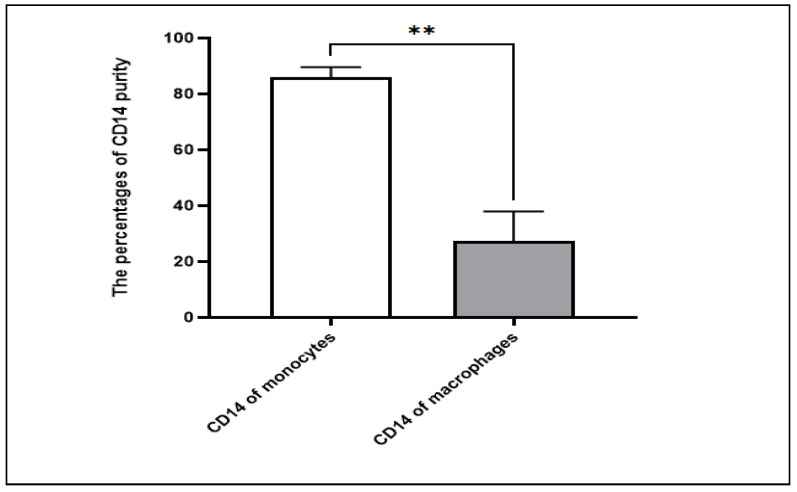
Comparison between the expression of CD14 on isolated monocytes and differentiated macrophages. The expression of CD14 cell-surface molecules decreased on differentiated macrophages after 7 days of culturing negatively selected monocytes, indicating the full conversion of monocytes to macrophages. *n* = 5. ** *p* value ≤ 0.01.

**Figure 4:**
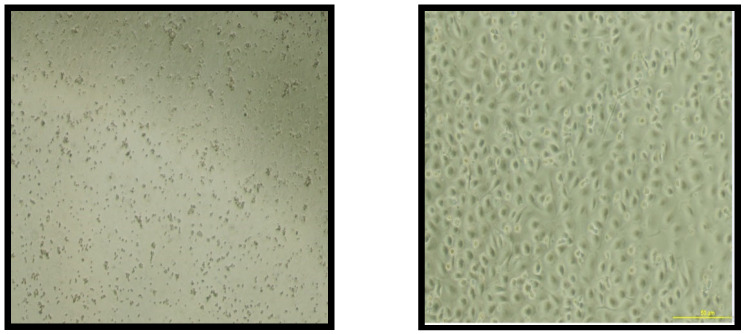
Morphological microscopic appearance of monocytes and macrophages. The figure on the left represents the shape of the isolated monocyte cells under the light microscope (×10) at day 1 immediately after magnetic separation. The figure on the right shows the monocyte-derived macrophages under the microscope (×10) after 7 days of monocyte culturing in the presence of GM-CSF. GM-CSF, granulocyte-macrophage colony stimulating factor.

After isolation, the monocytes were cultured for 7 days in a 12-well plate in the presence of required growth factor GM-CSF. The expression of CD14 cell-surface molecules after monocyte culturing significantly decreased compared to the expression at the start of the culture on day 1 (*p* = 0.0016), proving the differentiation of CD14 monocytes to become macrophages (Figure 3). In addition, according to the microscope pictures, the cells’ morphology levels on day 7 were compatible with the expected macrophage appearance (Figure 4).

### 3.3. Expression of Cell-Surface Molecules on Differentiated Macrophages

**Figure 5:**
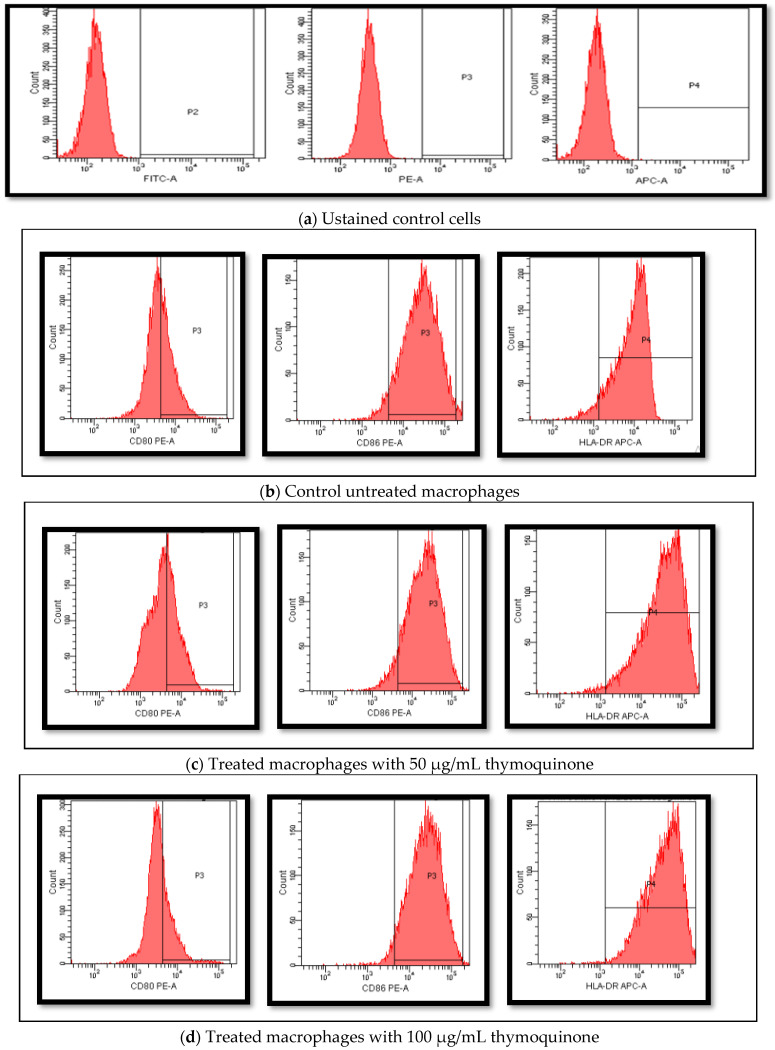
Flow cytometry histograms for the different detected cell-surface molecules on macrophages before and after treatment with TQ. A representation of flow data histograms for the cells’ gating setting and expression detection is shown. (**a**) Gate setting for the different fluorescent dyes used in the experiment (FITC, PE, APC). (**b**) Macrophages differentiated after 7 days’ culturing in the presence of GM-CSF. (**c**) Macrophages differentiated after 7 days’ culturing in the presence of GM-CSF and treated with 50 µg/mL TQ 24 h. (**d**) Macrophages differentiated after 7 days’ culturing in the presence of GM-CSF and treated with 50 µg/mL TQ 24 h. TQ, thymoquinone; FITC, fluorescein isothiocyanate ; PE, phycoerythrin; APC-A, allophycocyanin; CD80 PE-A, phycoerythrin monoclonal anti cluster of differentiation 80; CD86 PE-A, phycoerythrin monoclonal anti cluster of differentiation 86; HDLA-DR APC-A, allophycocyanin monoclonal anti cluster of differentiation human leukocyte antigens DR.

**Figure 6:**
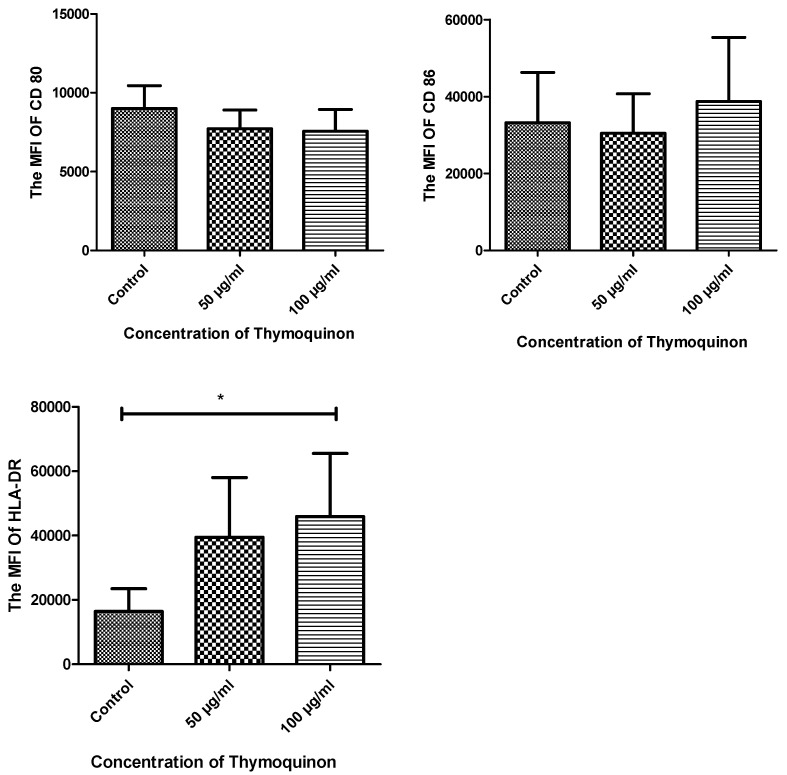
The MFIs of CD80, CD86 and HLA-DR expression in the three groups. The expression of cell-surface molecules on macrophages in different conditions (untreated macrophages (control), 50 μg/mL TQ-treated macrophages and 100 μg/mL TQ-treated macrophages) was checked using extracellular staining with CD80-PE, CD86-PE and HLA-DR APC. The TQ-treated cells showed no significant difference for both CD80 and CD86. However, a significant increase in the expression of HLA-DR compared to control was detected with the 100 μg/mL-treated cells. *p* value = 0.05 and *n* = 5. MFI, mean fluorescence intensity; CD80, cluster of differentiation 80; CD86, cluster of differentiation 8; HLA-DR, human leukocyte antigen DR isotype; CD86 PE-A, phycoerythrin monoclonal anti cluster of differentiation 86; HDLA-DR APC-A, allophycocyanin monoclonal anti cluster of differentiation human leukocyte antigens DR. * *p* value ≤ 0.05.

Macrophages were cultured as untreated and treated with different concentrations of TQ for 24 h. The macrophages’ cell-surface molecules CD80 and CD86 were detected. The results of CD80 showed no detectable differences (9007.8 ± 677.9 vs. 7717.6 ± 1262.3 vs. 2524.2 ± 1688.4) with a *p*-value of 0.842. Moreover, the expression of CD86 showed no significant differences (33,206.6 ± 13,083.7 vs. 28,429.0 ± 9080.2 vs. 40,489.6 ± 13,362.7) with a *p*-value of 0.313. Looking into the expression of HLA-DR, a significant increase in its expression was detected in treated cells with both concentrations compared to the control (14,659.0 ± 5855.1 vs 39,472.0 ± 18,565.6 vs. 45,905.3 ± 19,653.4) with a *p*-value of 0.05. Flow data and mean fluorescence intensity (MFI) comparison of the different markers are presented in Figure 5 and Figure 6.

### 3.4. Detection of Macrophages’ Secreted Cytokines Using ELISA

**Figure 7:**
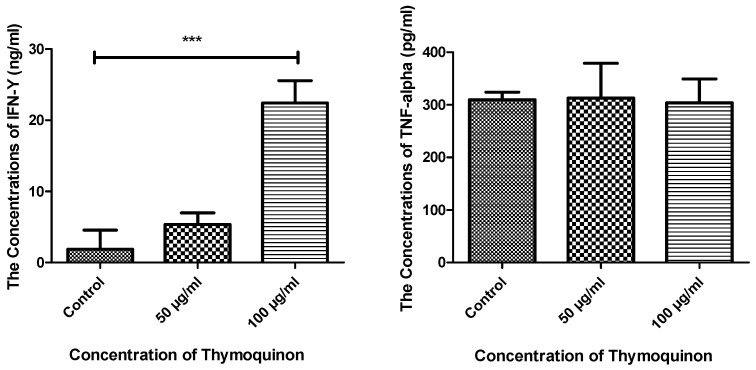
Comparison of the concentrations of IFN-γ and TNF-α secreted from macrophages in the three groups. The concentrations of both IFN-γ and TNF-α were detected in the different culture conditions as control, 50 µg/mL TQ-treated and 100 µg/mL TQ-treated cells. There was a significant difference in the concentration of IFN-γ secreted from the 100 µg/mL TQ-treated macrophages compared to control (*p* < 0.0001), while no changes were detected for TNF-α. *n* = 5. IFN-γ, interferon gamma; TNF-α, tumour necrosis factor alpha. *** *p* value ≤ 0.001.

The macrophage cytokines IFN-γ and TNF-α were examined after treatment with TQ. Data showed a significant effect of TQ at 100 μg/mL on the secretion of IFN-γ, as a higher concentration was recorded compared to the control, where *p* value < 0.0001. On the other hand, no changes were detected in the secretion of TNF-α between treated and control macrophages (Figure 7).

### 3.5. Macrophage Phagocytosis Ability Testing

**Figure 8:**
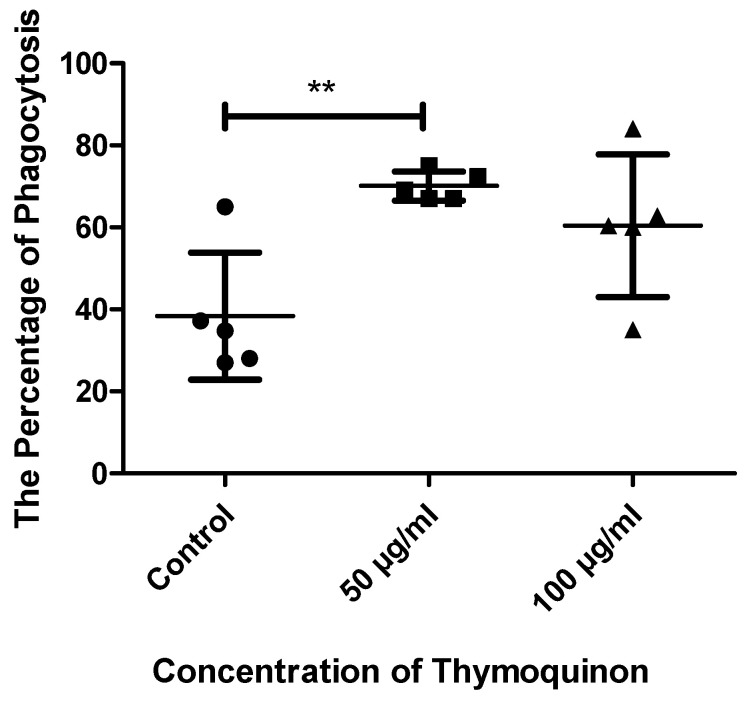
Comparison of the percentages of macrophage phagocytosis in the three groups. The phagocytosis abilities of the cells in different conditions (untreated macrophages, 50 µg/mL TQ-treated macrophages and 100 µg/mL TQ-treated macrophages were checked using flow cytometry to detect the percentage of macrophages that phagocyted FITC-conjugated *E. coli*. The results showed increased abilities of treated cells for phagocytosis, as significant results detected with the 50 µg/mL TQ showed that 68% of the cells phagocytosed *E. coli* compared to 41% of control cells. In addition, 58% of the 100 µg/mL TQ-treated cells phagocytosed *E. coli*. *n* = 5. ** *p* value ≤ 0.01.

Macrophage phagocytosis was tested by incubating cells with FITC fluorescent *E. coli*, and phagocytosis was checked using flow cytometry. The results showed significant differences in the treated macrophages, specifically in 50 µg/mL TQ, compared to the control (41 ± 16.57 vs. 68.25 ± 5.38 vs. 56.75 ± 15.56) with a *p*-value of 0.050, because 68% of the treated cells were shown to phagocyte *E. coli* compared to 41% of the untreated cells. The 100 µg/mL TQ also showed increased abilities for phagocytosis as the percentage of cells reached 56% (Figure 8). 

## 4. Discussion

Macrophages are a type of immune cell that originates from bone marrow monocytes that, in case of infection, migrate to the body tissues and differentiate into macrophages [21,22]; they play a defensive role against pathogens, such as microbes, and an essential role in homeostatic maintenance in the body by removing interior waste materials and repairing tissues [23]. The phagocytosis process is the primary mechanism of macrophages, allowing them to engulf different types of antigen and degrade them into pieces, then display the residual antigen peptides over the cell membrane to allow the adaptive system to recognize and attack the antigen [22]. However, although macrophages have the capacity to present antigens to T cells to activate adaptive immunity, they must first differentiate and express the antigens using surface molecules, such as CD80, CD86, and MHC II, required for interaction with T cells [4].

Medicinal herbs have been used to treat, prevent, and cure many human diseases since ancient times [24]; it is known that they can modify pathological and physiological processes [25]. One of the most common and important medicinal plants is *N. sativa*, or black seed. *N. sativa* seeds are used to enhance health and defend the body against diseases [15]. The main active chemical component present in *N. sativa* is TQ [16]. Studies on *N. sativa* with macrophages are limited and, for that reason, the aim of this study was to examine the effects of TQ, as the most important component of *N. sativa*, on the immunological properties and functionality of macrophage cells, one of the most important components of the immune system.

During inflammation, levels of immune modulators, such as cytokines, chemokines, leukotrienes, lytic enzymes, and nitric oxides, are elevated, and the anti-inflammatory properties of TQ on the immune system have been recorded in many studies. One study showed a preventive effect of TQ on the production of leukotrienes B4 and C4 in the blood due to the inhibitory effect of TQ on arachidonic acid transformation to 5-hydroxyeicosatetraenoic. In addition, the protective effect of TQ against bacterial infection in a rat model was recorded. TQ treatment decreases cytokine production and tissue damage and increases intestinal barrier action [26]. In the rat model, TQ was found to decrease interleukin-1β (IL-1β), IL-6, IFN-β, TNF-α and prostaglandin (PGE), which prevent pulmonary inflammation [27]. Decreasing these inflammatory mediators helps in minimizing alveolar macrophage and neutrophil damage caused by lytic enzymes and oxygen radicals [28]. It is suggested that TQ inhibition for leukotrienes and prostaglandins is a cause of inhibitory action on cyclooxygenase (COX), as TQ leads to the downregulation of NF-kβ and 1/AP1 needed for the activation of COX [29]. This effect on the cytokine and immune modulator expression affects both cellular and humoral responses. Other studies showed the important immune-enhancing effect of TQ on the maturation of DC, the cytotoxicity of NK, the activation of T cells, chemotaxis, and phagocytosis [26]. A study of RAW264 macrophage cell line using a component of *N. sativa* other than TQ showed an activating effect on the NF-kβ signaling pathway that caused enhanced cellular properties, including proliferation, phagocytosis and the secretion of TNF-α, IL-6 and NO [30]. Different study models, *N. sativa* components used, and dosage used are all factors that could lead to different conclusions for possible effects on the immune system.

In the study, the cell model used depended on monocyte-derived macrophages. The negative selection technique was used, and the purity of CD14-separated monocytes was around 83–89%. The data showed that, after monocytes were cultured for 7 days in the presence of GM-CSF, the CD14-FITC molecules showed a significant decrease in their expression compared to CD14-FITC in the isolated monocytes; this indicated that the monocytes had differentiated successfully into macrophages, which is consistent with other studies [31,32].

In monocyte-derived macrophages studies, different growth factors could be used to induce the differentiation of monocytes to either an M1 or M2 macrophage. Both GM-CSF and macrophage colony-stimulating factor (M-CSF) are used in vitro for this reason [1]. While GM-CSF is extensively produced during inflammation by active immune cells, M-CSF is normally circulating in adequate amounts to maintain the homeostasis of monocytes/macrophages. Stimulation of monocytes using GM-CSF induces an M1 macrophage subset that is proven to be involved in antibacterial/anticancer responses, while M-CSF induces an M2 macrophage subset that has a role in tissue repair and wound healing [33]. Both subsets are known to express different cell-surface molecules, as M1 expresses CD80 and CD86, while M2 expresses cluster of differentiation 163 (CD163) and cluster of differentiation 206 (CD206) [34]. In this study, as GM-CSF was used, CD80, CD86 and HLA-DR were selected.

CD80 and CD86 expression did not show any detectable difference between the treated and untreated cells. CD80 and CD86 are two important co-stimulatory molecules, part of the B7 family, and bridge the gap between the innate and adaptive immune responses [5]. As transmembrane proteins on the surface of APCs, CD80 and CD86 play a role in exhibiting both the recognition within the innate response and activation of the adaptive response. CD80 and CD86 are expressed by monocytes, macrophages, dendritic cells, and B cells [35]. Two signals are required for T-cell activation. The first signal is generated as a result of the interaction of the antigen–MHC complex with the receptors of T cells (TCR), while the second signal is generated by the interaction of CD80 or CD86 on APC with CD28 that is located on the T-cell surfaces [5,36]. The interaction between CD80 or CD86 with CD28 leads to T-cell activation, proliferation, and differentiation to stimulate the acquired system. They also help other cells, such as B cells and NK cells, to initiate an immunological response [37]. According to the results, a higher increase in the expression of co-stimulatory molecules and HLA-DR after GM-CSF was recorded, in agreement with previous studies [38]. However, TQ has no effect on either molecule, CD80 or CD86, at either of the concentrations used. On the other hand, HLA-DR (MHC II) expression showed a significant increase in treated macrophage cells, specifically with 100 μg/mL TQ. MHC II on macrophages is important in processing and presenting antigens to T cells and thus activating inflammatory responses. In addition, MHC II functions as a connector between macrophages and T cells in various inflammatory conditions [6]. Looking at the results, TQ has a positive effect on the expression of MHC II on macrophages, which means it can enhance the ability of macrophages to present antigens to T cells and thus provide the required first signal for T cell activation, enhancing its function as an APC. More studies are needed to examine the ability of treated macrophages to present antigens.

In this study, the ability of macrophages to secrete cytokines, such as IFN-γ and TNF-α, as important immunological mediators to orchestrate the immune response, was examined. Although it is well known that lymphoid origin cells, such as T cells and NK cells, are the principal producers of IFN-γ, there is some evidence suggesting a possible role of macrophages and other APCs in the production of this potent cytokine [39]. Some studies examined the secretion of IFN-γ by monocyte-derived macrophages and demonstrated the presence of IFN-γ after stimulation with a combination of either IL-12 and IL-18 or MCSF [40,41]. In the present study, the cells were stimulated with GM-CSF, and the secretion of IFN-γ was detected. IFN-γ is one component of immunological response that is important in both cell autoactivation and the activation of nearby cells [42]. It has been suggested that IFN-γ produced by APCs has an important autocrine action on the cells themselves and in the activation of nearby cells, while T-cell IFN-γ has paracrine action in adaptive immunity [39]. The present data showed a significant effect of 100 μg/mL TQ on the secretion of IFN-γ, but not TNF-α. A study by Maroua and her colleagues investigated the effect of TQ treatment on classically activated macrophages with or without necrotic Jurkat cell lysate (NecrJCL). They found that combining TQ with NecrJCL increased the production of interleukin-2 (IL-2), IL-6, interleukin-17 (IL-17), IFN-γ, TNF-α and phagocytosis. They concluded that TQ has a positive effect on NecrJCL-pulsed macrophages [43], which is in agreement with the present data that point to the possible enhancement of macrophage activity during innate immunity.

On the other hand, TNF-α is produced by macrophages, regulating many of its functions and affecting other cells and organs [44]. It is considered an inflammatory cytokine and is known to be one the first cytokines released during infection. It is responsible for the acute phase of immunity, leading to an increased infiltration of cells to the site of infection by increasing vasodilation and the permeability of blood vessels. In addition, it orchestrates the release of other chemokines to elevate the immune response. TNF-α is also a potent activator for c-reactive protein and other mediators during inflammation [45]. With the TQ concentrations used, no changes in the secretion of TNF-α were detected. In a study performed on murine macrophage-like RAW 264 cells, treatment with TQ showed an anti-inflammatory effect on the production of TNF-α caused by the suppression of the NF-kB pathway [29]. Another study checked the level of a number of inflammatory molecules, including TNF-α in an *E. coli*-challenged animal, and also detected an anti-inflammatory effect of TQ on the secreted TNF-α, suggesting a role for TQ in the modulation of the immune response as an anti-inflammatory agent [46]. More detailed studies are required to confirm the effect of TQ on these cells.

Phagocytosis is one of the most important features of the immunological properties of macrophages [47]. This was examined by looking into the abilities of macrophages treated with TQ to phagocytose fluorescent *E. coli* and the percentage of cells that had taken up and engulfed the bacteria. The data showed a significant positive difference for treated cells. The macrophage phagocytosis results indicate that TQ has a positive effect on the cells’ abilities in an extremely important innate defensive mechanism. This result agrees with previous work by Barnawi and colleagues, where they also found that TQ enhanced the phagocytosis of bacteria and apoptotic cells by macrophages. They pointed, in their study, to the positive role of TQ in the modulation of the sphingosine-1-phosphate (S1P) system. Changes associated with inflammation and oxidative stress may affect S1P and thus lead to the dysfunction of macrophage phagocytosis [48]. This study points to a possible positive effect of TQ on the functionality of macrophages.

The results of this study support the possibilities of immunostimulatory action of TQ on immune cells. A study on the J774A murine macrophage cell line showed an increase in cell proliferation after *N. Sativa* treatment, which is supported by another study that recorded increased phagocytosis [49]. More detailed studies are needed on immune cells, investigating their mechanism of action and subsequent alterations associated with the treatment with TQ.

## 5. Conclusions

In conclusion, the effect of thymoquinone on macrophages differentiated from monocytes was studied. The expression of important cell-surface molecules, cytokine secretions, and phagocytotic abilities were examined. In general, the study supports the possibility that TQ treatment enhances macrophage activity. This was demonstrated by the significant increase in MHC II molecules on treated macrophages as well as an increase in IFN-γ concentration and the phagocytotic abilities of the cells after treatment. This may indicate that TQ improved the performance of macrophages during innate immunity via phagocytosis and the processing and presentation of antigenic materials on their MHC II molecules, thus enhancing the antigen presentation by macrophages to T cells. More studies in this field are needed, as TQ could be an important therapeutic candidate for the enhancement of the immunological properties of individuals.

## Data Availability

Not applicable.
